# Short stature, digit anomalies and dysmorphic facial features are associated with the duplication of miR-17 ~ 92 cluster

**DOI:** 10.1186/1755-8166-7-27

**Published:** 2014-04-16

**Authors:** Morteza Hemmat, Melissa J Rumple, Loretta W Mahon, Charles M Strom, Arturo Anguiano, Maryam Talai, Bryant Nguyen, Fatih Z Boyar

**Affiliations:** 1 Cytogenetics Department, Quest Diagnostics, 33608 Ortega Hwy, San Juan Capistrano, California 92675, USA; 2 Banner Child Neurology, 5310 W Thunderbird Rd, Ste 301, Glendale, Arizona 85306, USA; 3 Quest Diagnostics, 8401 Fallbrook Avenue, West Hills, Los Angeles, California 91304, USA

**Keywords:** miRNA, MIR17HG, miR-17 ~ 92, GCP5, Digital anomaly, Skeletal defects

## Abstract

MicroRNAs (miRNAs) are key regulators of gene expression, playing important roles in development, homeostasis, and disease. Recent experimental evidence indicates that mutation or deregulation of the *MIR17HG gene* (miR-17 ~ 92 cluster) contributes to the pathogenesis of a variety of human diseases, including cancer and congenital developmental defects. We report on a 9-year-old boy who presented with developmental delay, autism spectrum disorder, short stature, mild macrocephaly, lower facial weakness, hypertelorism, downward slanting palpebral fissures, brachydactyly, and clinodactyly. SNP-microarray analysis revealed 516 kb microduplication at 13q31.3 involving the entire *MIR17HG* gene encoding the miR-17 ~ 92 polycistronic miRNA cluster, and the first five exons of the *GPC5* gene. Family study confirmed that the microduplication was maternally inherited by the proband and one of his five half-brothers; digit and other skeletal anomalies were exclusive to the family members harboring the microduplication.

This case represents the smallest reported microduplication to date at 13q31.3 and provides evidence supporting the important role of miR-17 ~ 92 gene dosage in normal growth and skeletal development. We postulate that any dosage abnormality of *MIR17HG*, either deletion or duplication, is sufficient to interrupt skeletal developmental pathway, with variable outcome from growth retardation to overgrowth.

## Background

MicroRNAs (miRNAs) are single-stranded small molecules of approximately 22 to 24 nucleotides that are frequently expressed in clusters. They play an important role in the post-transcriptional control of gene expression by binding to complementary sites within target mRNAs, thereby mediating their degradation or repressing translation [1-3]. *MIR17HG*, the host gene for the miR-17 ~ 92 cluster, encodes for six individual miRNAs: *MIR17, MIR18A, MIR19A, MIR20A, MIR19B1* and *MIR92A1*. The miR-17 ~ 92 miRNA cluster is located on human chromosome 13q31.3, in a genomic region that is frequently amplified in lymphomas and other cancers. The mature miRNAs encoded by this locus are expressed in high amounts in cancer cells [4,5]. This cluster and its paralogues have important roles in cancers because of their ability to repress expression of many tumor-associated proteins [6-14]. Overexpression promotes cell proliferation and reduces apoptosis by regulating cell cycle progression [15-17].

The miR-17 ~ 92 cluster also plays an important role in normal growth and skeletal development. Transcription of this cluster is activated by both *MYC (C-MYC)*[18,19] and *MYCN (n-MYC)* oncogene [20]*. MYCN* regulates *MIR17HG* which itself involves in regulation of transforming growth factor beta (TGFB) and sonic hedgehog (SHH) pathways which have critical role in skeletal development and limb formation [20]. Reduction of miR-17 ~ 92 expression, or that of its paralogues, results in smaller embryos and postnatal death due to hypoplastic lungs and ventricular septal defects in mice [21]. While *MYCN* mutations or deletions cause autosomal dominant Feingold syndrome 1 with variety of abnormalities, heterozygous deletions of *MIR17HG* cause Feingold syndrome 2 with only skeletal anomalies and growth defects [20]. Three patients with germline microdeletions of the chromosomal region 13q31.3, including the miR-17-92 cluster and first 5 exons of the *GPC5* gene are presented with Feingold syndrome 2 [20]. In support of the hypothesis of MIR17HG having a major role in their patients’ abnormal phenotype, de Pontal and colleagues described two individuals with defective GPC5 gene expression who nevertheless had a normal phenotype [20]. Additionally, transgenic mice hemizygous for miR-17 ~ 92 deletion (with no involvement of *GPC5*) showed severe abnormalities in their digits but no change in GPC5 expression [21]. This suggests that GPC5 is not critical for normal skeletal development. However, GPC5 deletion or mutations may still contribute to certain clinical features observed in most patients with deletions involving both *MIR17HG* and *GPC5* genes.

Interestingly, duplication of the miR-17 ~ 92 and *GCP5* locus has also been described in patients with digit abnormalities. Kannu and colleagues recently reported a germline 912-kb duplication at 13q31.3, encompassing miR-17 ~ 92 and *GPC5*, in a patient presenting with a post-axial polydactyly type A and overgrowth [22]. Here we describe a case involving the smallest miR-17 ~ 92 microduplication reported to date. Affected family members exhibited multiple skeletal abnormalities, including digit and facial anomalies.

## Case presentation

### Case report

**Figure 1:**
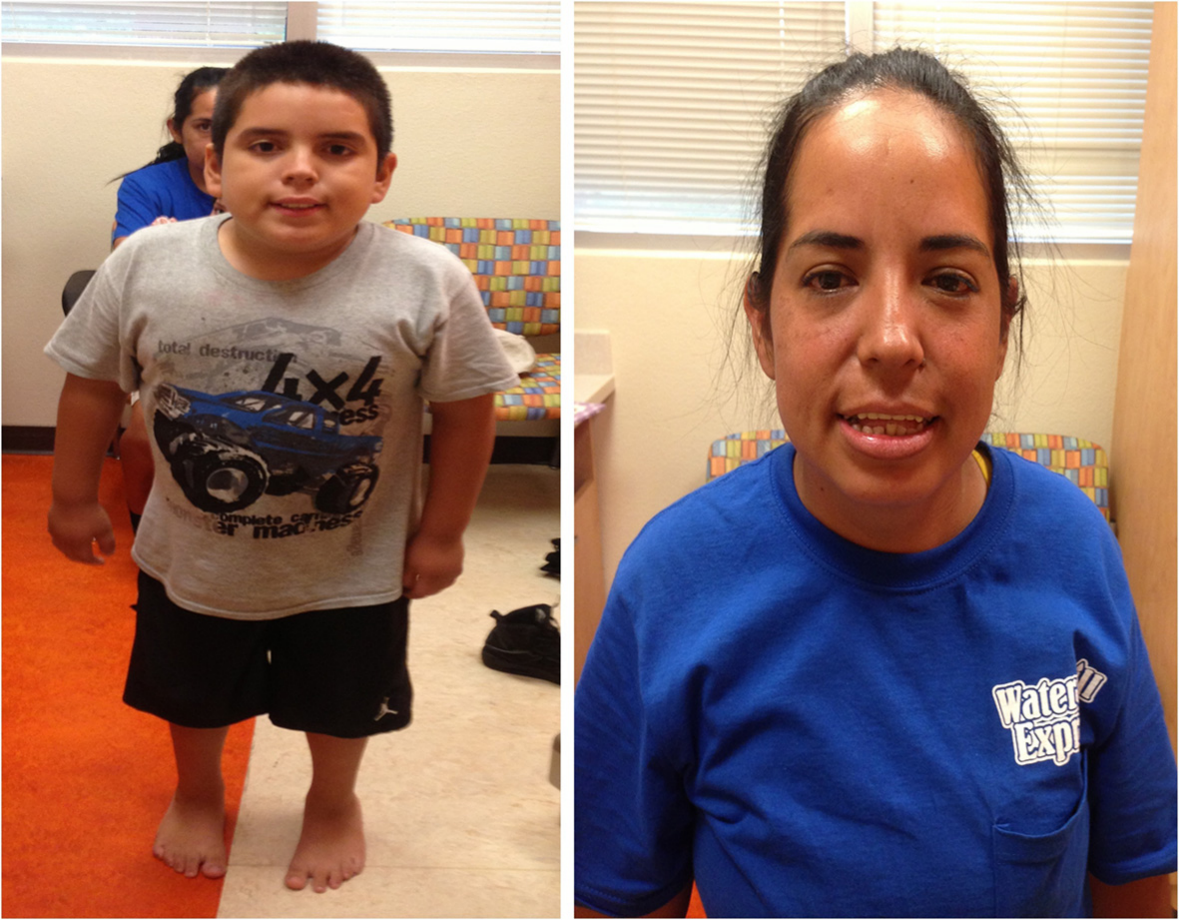
**Photographs of proband and his mother.** Proband (left) and his mother (right) showing lower facial weakness, hypertelorism, and downward slanting palpebral fissures.

**Figure 2:**
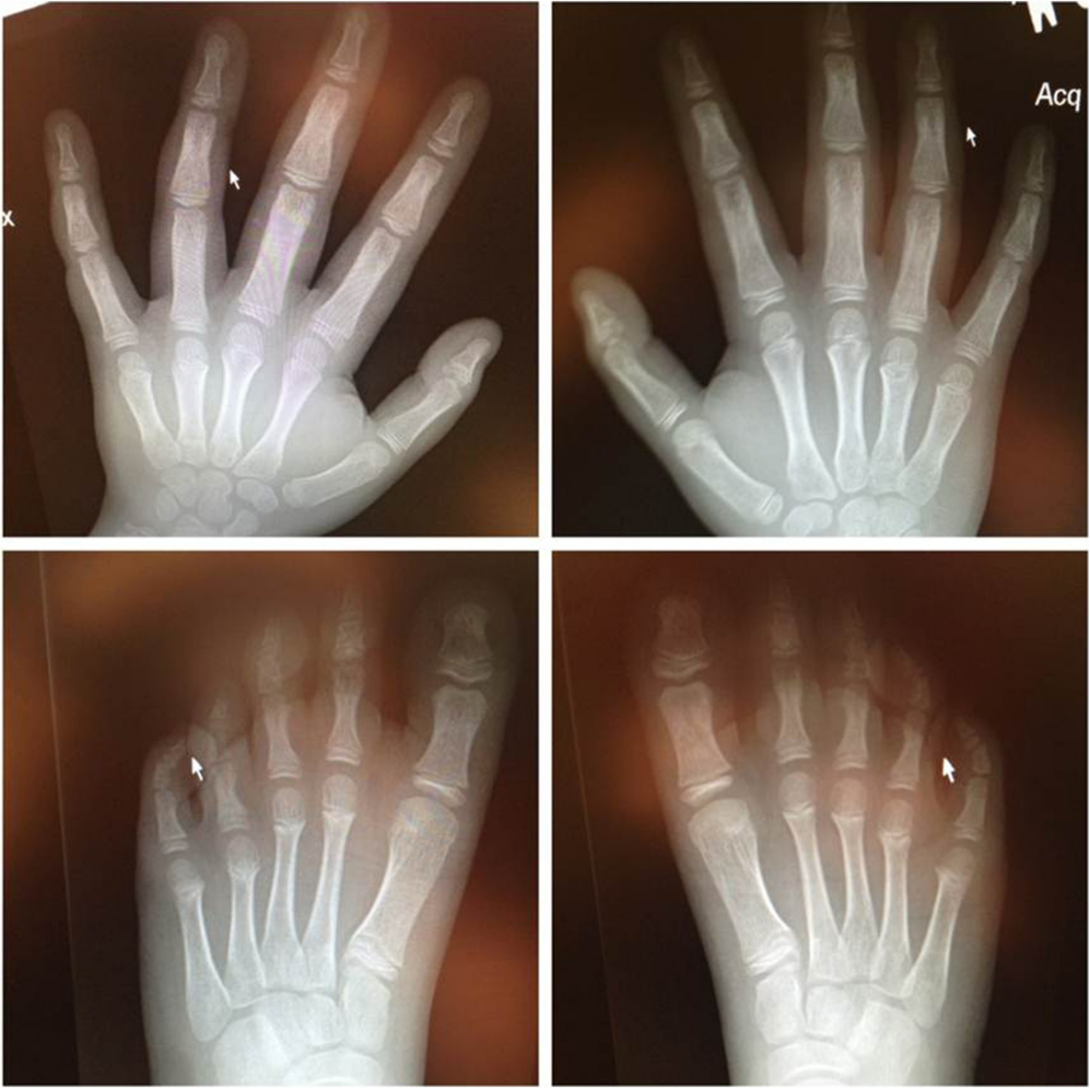
**Radiographs showing digit anomalies of the proband.** Anomalies included brachydactyly, wide nail beds, deviation of fourth digits to the ulnar side, radial displacement of thumbs, and clinodactyly of the fourth and fifth digit tarsal.

The proband presented at 9 years of age with developmental delay, autism spectrum disorder, speech delay, attention deficit hyperactivity disorder, and a variety of physical abnormalities: short stature (10^th^ percentile for the proband and <1^st^ percentile for the mother), mild macrocephaly in relation to body size, metacarpal and metatarsal anomalies, flat feet, lower facial weakness, hypertelorism, downward slanting palpebral fissures, frequent dental caries, and digit anomalies including brachydactyly, wide nail beds, deviation of fourth digits to the ulnar side, radially displaced thumbs, and mild clinodactyly (Figures 1 and 2). The hand breath, hand length, and hand circumference were <1% for age and gender for both the mother and proband. The proband was born at full term via vaginal delivery to a G4P4A0L4 25-year-old mother without any known maternal complications or infections. Birth weight (3,580 grams) was normal. No prenatal, perinatal, or postnatal complications were noted, and he was discharged on the second day of life. He first walked at 12 months but did not speak words until 3 years. Interventions included speech and physical therapy, an individualized education plan, and special education for diagnoses of developmental delay and autism spectrum disorder.

**Figure 3:**
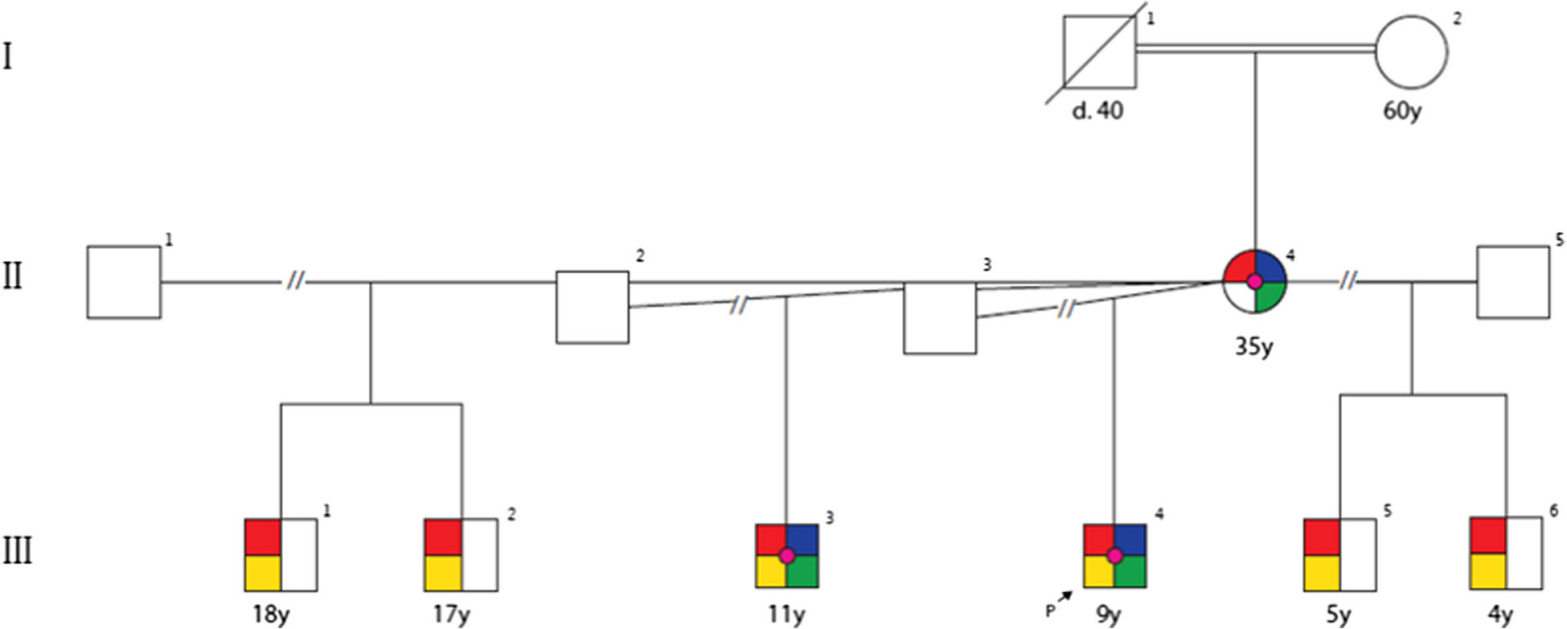
**Pedigree: The proband (III4) has five half-brothers (III1, III2, III3, III5 and III6) resulting from his mother’s four different marriages.** The proband (III4), his mother (II4) and one of his half-brothers (III3) harboring a 516 kb duplication at 13q13.3 (red small circle) showed dysmorphic facial features, lower facial weakness, and relative macrocephaly (blue square) as well as digit anomalies including clinodactyly, brachydactyly, and short stature (green square). Developmental delay (red square) and autism (Yellow Square) were observed in all six brothers. The mother showed all of the above abnormalities except autism.

The proband had five half-brothers resulting from his mother’s four different marriages (Figure 3). All were examined except two (18 and 17 years old), who were not available (living in Guatemala) but were reported to exhibit developmental delay and autism. One of the half-brothers (11 years old) and mother showed similar clinical features as the proband, although the mother did not have a diagnosis of autism. The other two examined half-brothers (4 and 5 years old) had normal hand breath, hand length, and hand circumference measurements (at the 50 percentile for their ages), with no evidence of polydactyly or syndactyly. However, all six brothers had autism and developmental delay.

### Methods

Genomic DNA was extracted from whole blood using the Gentra Puregene kit (Qiagen-Sciences, Maryland, USA). Microdeletion/microduplication screening was performed for the proband, his mother, and available half-brothers using an SNP-array platform (CytoScan HD; Affymetrix, Santa Clara, CA), following the manufacturer’s instruction. The CytoScan HD array has 2.67 million probes, including 1.9 million copy number probes and 0.75 million SNP probes. Array data were analyzed using the Chromosome Analysis Suite (ChAS) (Affymetrix, Inc.) software v 2.0.

### Results

**Figure 4:**
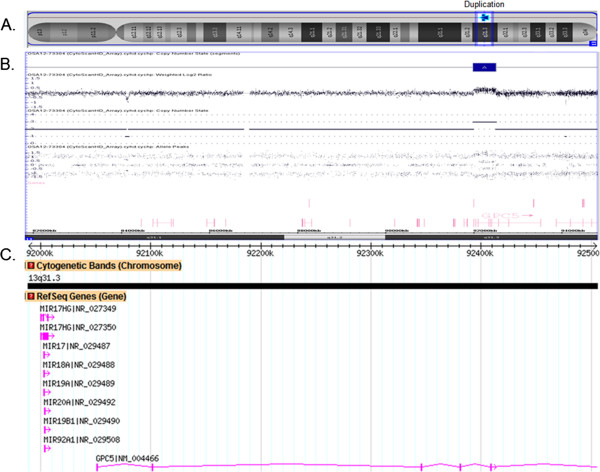
**Duplication of 13q31.3. A**. Chromosome 13 with duplication at 13q31.3. **B**. Proband SNP-array results, including the log2 ratio, weighted log2 ratio, copy number state, and the genes at the duplicated region. **C**. Database of genomic variants showing 515.6 kb duplication at chromosome 13, position 91,989,261 to 92, 504,857, including miR-17 ~ 92 and five exons of the *GCP5* gene.

This analysis detected a microduplication of 516 kb at 13q31.3 (Figure 4), which encompassed the miR-17 ~ 92 cluster genes (*MIR17, MIR18A, MIR19A, MIR20A, MIR19B1, MIR92A1*) and the first five exons of the *GPC5* gene. The duplication extended from 91,989,261 to 92,504,857 to 15,433,672 bp (UCSC genome Browser; http://genome.ucsc.edu/; hg19 release). FISH results suggested tandem duplication and excluded an unbalanced translocation or insertion. The duplication was also detected in our proband, his mother, and one of his five half-brothers, segregating with short stature, clinodactyly, brachydactyly, short digits, and dysmorphic facial features with lower facial weakness. Developmental delay and autism were observed in all six brothers and thus were not exclusive to those harboring the 13q31.3 duplication, although they might have been maternally inherited (Figure 3).

Additionally, SNP array analysis in the proband’s mother showed a number of copy neutral regions of allelic homozygosity (ROH). The additive amount of ROH across the genome that span >5 Mb totaled approximately 128 Mb. The implications of ROH are unclear at present. In theory, when the degree of homozygosity is significant, there is an increased risk for recessive Mendelian disorders.

## Discussion and conclusions

Multiple studies have indicated that the gene content of the 13q31.3 region is associated with normal growth and skeletal development. In this respect de Pontual et al. and colleagues [20] stated that deletion of miR-17 ~ 92 plays a major role in skeletal defects and ruled out any involvement of the *GCP5* gene. In contrast, Kannu et al. recently reported a case with a larger microduplication but very similar gene content to our case [22]. Their patient had a 909-kb microduplication encompassing the same genes, including the entire miR17 ~ 92 cluster and first five exons of the *GPC5* gene, exhibited post-axial polydactyly type A and overgrowth. The authors proposed that deletion of the miR-17 ~ 92 cluster genes may result in haploinsufficiency and growth retardation, while duplication results in overexpression and overgrowth; Van der Zwaag et al. also reported a larger (5.6 Mb) duplication, which included the *MIR17HG* and *GPC5* genes, in a patient with postaxial polydactyly A2 [23]. However, our case contradicts their hypothesis, since the affected family members had a similar duplication but exhibited features of growth retardation such as short stature, brachydactyly, and short digits. Our finding is also supported by Shan et al. [24], who developed a line of transgenic mice overexpressing pre-miR17 by introducing a fragment of cDNA harboring four copies of the pre-miR-17. The transgenic mice overexpressed miR17 and exhibited overall growth retardation, smaller organs, and greatly reduced hematopoietic cell lineages.

While recent reports strongly support the involvement of miR-17 ~ 92 in skeletal development, the role of *GCP5* gene has been controversial. No abnormal phenotype has been observed in transgenic mice and two control individuals harboring heterozygous deletion of *GPC5* only [20], while the expression of *GPC5* gene has been well characterized in the developing mouse limb bud, where it is highly expressed in the mesenchymal condensation of the digits [25].

This report represents the smallest microduplication involving the *MIR17HG* and *GPC5* genes described to date and provide additional evidence to support the complex role of miR-17 ~ 92 gene dosage in normal growth and skeletal development. The skeletal anomalies observed in affected family members of our patient appear to be inconsistent with other reported cases with duplication. It is possible that either deletion or duplication of miR-17 ~ 92 cluster can interrupt skeletal developmental pathways, with variable expression from growth retardation to overgrowth. Further investigations, such as expression studies, are needed to clarify the exact role of miR-17 ~ 92 in skeletal development.

## Ethical approval and consent

These studies were performed on anonymized samples received in the clinical laboratory and thus were exempted from the requirement for consent by an opinion for the Western Institutional Review Board. However, written informed consent was obtained from the parents for publication of this case report. A copy of the written consent is available for review by the Editor-in-Chief of this journal. The microarray studies were performed on anonymized samples provided to the cytogenetic department, Quest Diagnostics Inc.

## Abbreviations

miRNA: Micro RNA; MIR17HG: The micro RNA host gene for the miR-17 ~ 92 cluster.

## Competing interests

The authors declare that they have no competing interest.

## Authors’ contributions

MH, First authors; performed analysis, interpretation of the results, drafting and finalizing the manuscript. MJR and LWM participated in writing the case description and gathering the family information. MT performed the SNP-microarray assay. BN constructed the patient’s family pedigree. All authors read and approved the final manuscript.
